# Health Impacts of Pre-eclampsia: A Comprehensive Analysis of Maternal and Neonatal Outcomes

**DOI:** 10.3390/medicina60091486

**Published:** 2024-09-12

**Authors:** Flavius George Socol, Elena Bernad, Marius Craina, Simona-Alina Abu-Awwad, Brenda-Cristiana Bernad, Ioana Denisa Socol, Ahmed Abu-Awwad, Simona Sorina Farcas, Daniel Laurențiu Pop, Daniela Gurgus, Nicoleta Ioana Andreescu

**Affiliations:** 1 Doctoral School, “Victor Babeş” University of Medicine and Pharmacy, Eftimie Murgu Sq. No.2, 300041 Timisoara, Romania; george.socol@umft.ro (F.G.S.); bernad.brenda@umft.ro (B.-C.B.); ioana.socol@umft.ro (I.D.S.); daniellaurentiupop@yahoo.com (D.L.P.); 2 Ist Clinic of Obstetrics and Gynecology, “Pius Brinzeu” County Clinical Emergency Hospital, 300723 Timisoara, Romania; craina.marius@umft.ro (M.C.); alina.abuawwad@umft.ro (S.-A.A.-A.); 3 Department of Obstetrics and Gynecology, Faculty of Medicine, “Victor Babeş” University of Medicine and Pharmacy, 300041 Timisoara, Romania; 4 Center for Laparoscopy, Laparoscopic Surgery and In Vitro Fertilization, “Victor Babeş” University of Medicine and Pharmacy, 300041 Timisoara, Romania; 5 Center for Neuropsychology and Behavioral Medicine, “Victor Babeş” University of Medicine and Pharmacy, 300041 Timisoara, Romania; 6 Department XV—Discipline of Orthopedics—Traumatology, “Victor Babeş” University of Medicine and Pharmacy, 300041 Timisoara, Romania; ahm.abuawwad@umft.ro; 7 Research Center University Professor Doctor Teodor Sora, Faculty of Medicine, Discipline II Orthopedics, “Victor Babeş” University of Medicine and Pharmacy, 300041 Timisoara, Romania; 8 Department of Microscopic Morphology—Genetics, Center of Genomic Medicine, “Victor Babeş” University of Medicine and Pharmacy, Eftimie Murgu Sq. No.2, 300041 Timisoara, Romania; farcas.simona@umft.ro (S.S.F.); andreescu.nicoleta@umft.ro (N.I.A.); 9 Department of Balneology, Medical Recovery and Rheumatology, Family Discipline, Center for Preventive Medicine, “Victor Babeș” University of Medicine and Pharmacy, 300041 Timisoara, Romania; gurgus.daniela@umft.ro

**Keywords:** pre-eclampsia, maternal health, neonatal outcomes, long-term health effects, proteinuria, sFlt-1, PlGF, biochemical markers, premature birth, low birth weight

## Abstract

*Background and Objectives*: Hypertensive disorders, particularly pre-eclampsia, pose significant risks during pregnancy, affecting both maternal and neonatal health. The study aims to analyze short- and long-term health implications for mothers and their children, comparing those with pre-eclampsia to those without, to improve understanding of risk factors, diagnostic markers, and outcomes. *Materials and Methods*: This retrospective observational study involved 235 patients, 98 with pre-eclampsia and 137 without, monitored from 2015 to 2018 at the Obstetrics and Gynecology Department of the “Pius Brînzeu” Emergency County Clinical Hospital in Timișoara, Romania. *Results*: Women with pre-eclampsia were older, had higher BMIs, and more frequently had a family history of pre-eclampsia, hypertension, and diabetes. They also had lower educational and socioeconomic levels and fewer prenatal visits. Biochemical markers such as higher proteinuria, elevated sFlt-1, and lower PlGF were significant in diagnosing pre-eclampsia. Short-term maternal complications like eclampsia, HELLP syndrome, and acute kidney injury were more prevalent in the pre-eclampsia group. Neonatal outcomes included higher rates of preterm birth, low birth weight, and NICU admissions. Long-term mothers with a history of pre-eclampsia had higher incidences of chronic hypertension, cardiovascular disease, kidney problems, diabetes, and mental health disorders. Their children faced increased risks of neuropsychological delays, chronic respiratory issues, behavioral disorders, learning difficulties, and frequent infections. *Conclusions:* The study highlights the significant short- and long-term health impacts of pre-eclampsia on both mothers and their children. Early monitoring, intervention, and comprehensive management are crucial in mitigating these risks. These findings underscore the need for personalized care strategies to improve health outcomes for affected individuals.

## 1. Introduction

During pregnancy, hypertensive disorders, such as elevated blood pressure, are common, with a prevalence of 5–10% [1]. These disorders can affect the health of both the mother and the unborn child. It is crucial to closely monitor and control blood pressure levels, especially for pregnant women with a pre-existing condition that raises the risk of developing pre-eclampsia.

When hypertension during pregnancy becomes a long-term condition, it often comes with serious complications such as eclampsia. Additionally, it is commonly associated with premature delivery, which occurs before the pregnancy reaches the 37-week mark [2]. Furthermore, chronic high blood pressure can lead to low birth weight (weighing less than 2500 g). Continual medical monitoring is essential to secure the best outcomes for the mother and the baby. [3]. The main aim is to protect the mother’s health and support the fetus’s growth to reach a healthy birth weight and be carried to full term.

There are long-term health effects of high blood pressure during pregnancy that go beyond the time of pregnancy. Mothers who experience high blood pressure during pregnancy have a significantly higher likelihood of developing cardiovascular disease [4]. Previous research showed that having high blood pressure during the first pregnancy was linked to an 80% higher chance of developing cardiovascular disease later in life [5].

Pre-eclampsia is characterized by the emergence of hypertension after the 20th week of pregnancy, accompanied by signs of dysfunction in maternal organs or the uteroplacental unit or by the presence of protein in the urine [6]. Pre-eclampsia is associated with a four times greater risk of having a heart attack within ten years after giving birth [7]. This underscores the importance of ongoing cardiovascular monitoring and preventive care for these women to improve long-term health outcomes [8].

The purpose is to provide a detailed understanding of how pre-eclampsia affects the mother and child at various stages of life.

This study was conducted on the increased danger of lasting health problems for mothers who have a history of pre-eclampsia. This includes things like chronic hypertension, cardiovascular diseases, kidney issues, diabetes, and mental health worries. It also looked at the possible higher risks for infants born to mothers who had pre-eclampsia which can involve neurodevelopmental delays, chronic respiratory issues, behavioral disorders, learning difficulties, and more infections.

This research focused on examining the differences in demographic, medical, and biochemical factors between women suffering from pre-eclampsia and those who do not have this condition. It also aimed to assess the effects on health for mothers and their babies in the short and long term. The study’s goals were to improve understanding about what risks, signs for diagnosis, and consequences come with pre-eclampsia, emphasizing how critical it is to monitor and intervene promptly to improve outcomes for mother–child health.

## 2. Materials and Methods

### 2.1. Study Design and Population

This retrospective observational study involved 235 patients: 98 with pre-eclampsia (pre-eclampsia group—PEG) and 137 without pre-eclampsia (control group—CG). Pregnant women were enrolled in the study at the time of their diagnosis of pre-eclampsia, which is typically after 20 weeks of gestation.

Pre-eclampsia is diagnosed when a woman, after 20 weeks of gestation, exhibits hypertension, defined as a systolic blood pressure of ≥140 mmHg or a diastolic blood pressure of ≥90 mmHg on two occasions at least four hours apart, along with proteinuria (≥300 mg per 24 h urine collection, a protein/creatinine ratio ≥ 0.3, or a dipstick reading of 1+ or greater). In the absence of proteinuria, pre-eclampsia can also be diagnosed if hypertension is accompanied by thrombocytopenia (platelet count < 100,000/µL), renal insufficiency (serum creatinine > 1.1 mg/dL or doubling of creatinine), impaired liver function (elevated transaminases), pulmonary edema, or new-onset cerebral or visual disturbances [6]. All patients were treated with doses of Nifedipine and Dopegyt (with the active ingredient: methyldopa). From 2015 to 2018, the patients were monitored throughout their pregnancies at the Obstetrics and Gynecology Department of the “Pius Brînzeu” Emergency County Clinical Hospital in Timișoara, Romania. All patients received regular follow-up and monitoring during pregnancy at the hospital where the study was conducted. It is essential to mention that specialized medical personnel established the long-term diagnoses of both mothers and children. This represents a fundamental concept and an exceedingly important factor in determining the prognosis for both the health of mothers and their children. Exclusionary diagnoses are made at multiple points throughout the study. Primarily, exclusions are identified before pregnancy during the initial assessment, ensuring that participants meet the necessary criteria for inclusion. However, additional exclusion criteria may be applied during pregnancy if new relevant conditions could impact the study’s outcomes. Finally, any emerging diagnoses that align with the exclusion criteria are also considered during follow-up, and these participants may be excluded from further analysis. This multi-phase approach ensures that the study population remains as relevant and homogenous as possible for accurate and meaningful results. The medical analyses provided in this study were collected at the time of the pregnant women’s admission to the hospital for childbirth.

### 2.2. Inclusion and Exclusion Criteria

We have established specific criteria for participant inclusion and exclusion to ensure the study results remain unbiased based on potential factors. Inclusion criteria include pregnant individuals over 18 years old with a confirmed diagnosis of pre-eclampsia for the pregnant women in Group 1, access to complete medical records, and willingness to provide informed consent. These documented medical conditions appeared in the mother at a gestational age of at least 20 weeks at enrollment in the study; the mother was able to give her consent for follow-up and adherence to the study protocols, the mother has a single pregnancy, constant access to prenatal care throughout, with no history of previous miscarriages of pregnancy and no prior incidents or diagnoses of COVID-19 infection.

Similarly, exclusion criteria have been defined to ensure the study’s integrity and reliability. These criteria include individual patients with inadequate access to prenatal care during pregnancy, incomplete or missing medical records, and those diagnosed with infectious diseases such as Hepatitis B Virus (HBV), Hepatitis C Virus (HCV), Human Immunodeficiency Virus (HIV), or Acquired Immunodeficiency Syndrome (AIDS). Additionally, pregnant women with a documented history of cancer, diagnosed mental health disorders, or facing substance abuse challenges, whether related to drugs or alcohol, were excluded from the study; patients with fetal genetic anomalies or other genetic abnormalities were excluded from the analysis because many of these cases are likely to present long-term complications.

### 2.3. Statistical Analysis

The statistical analysis was conducted using MedCalc, an advanced tool for statistical processing in the biomedical field. For the demographic and clinical characteristics of the study population, we employed descriptive statistics, including mean and standard deviation (SD) for continuous variables (such as age, systolic and diastolic blood pressure, proteinuria levels, serum creatinine, and serum uric acid levels) and percentages for categorical variables (for example, urban/rural residence, smokers). The independent samples *t*-test was used to compare the means of continuous variables between the two groups. The *p*-value associated with each comparison was reported to determine the statistical significance of the observed differences. A significance threshold of 0.05 was considered for all tests. We used the Chi-squared test to evaluate the percentage differences for categorical variables. Correlation analysis was performed to identify and assess the strength of the association between pre-existing medical conditions and the severity of pre-eclampsia. Correlation coefficients and associated *p*-values were provided to highlight statistically significant relationships. This methodological approach allowed us to offer a rigorous and detailed analysis of the data, contributing to a deeper understanding of the demographic, clinical, and obstetric profiles of women with hypertension during pregnancy in the specific context of this medical setting.

### 2.4. Ethical Considerations

This study received approval from the hospital’s ethics committee with approval number 72/28 in June 2021. Adhering to ethical guidelines, every participant gave informed consent before participating in the research. This involved their agreement to participate actively in the survey, authorization to collect blood samples for analysis, and permission to compile their personal and health information.

Moreover, the study took extra measures to safeguard the privacy and confidentiality of all individuals involved. All of the collected data were carefully anonymized, ensuring no personal identifiers were attached to the information. This stringent approach further underscored the study’s commitment to maintaining the highest ethical standards throughout its course.

## 3. Results

The study participants were divided into pre-eclampsia (PEG) and control (CG) groups.

**Figure 1:**
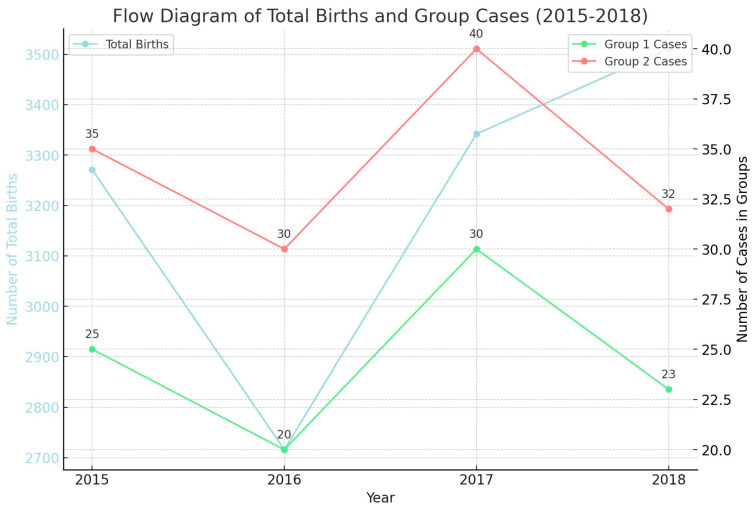
Flow diagram of total births and group cases.

Figure 1 illustrates the total number of births from 2015 to 2018 alongside the distribution of cases in two groups (Group 1 and Group 2). The total births, represented by a light blue line, show a fluctuation, with a dip in 2016 followed by an increase, peaking in 2018. Group 1, shown by the green line, exhibits minor fluctuations with a peak in 2017, while Group 2, represented by the red line, shows a more pronounced increase, peaking in 2017 before slightly decreasing in 2018. The graph highlights the trends and variations in case distribution relative to total births, providing insights into potential demographic or medical shifts over the period studied.

Table 1 systematically presents the baseline data of these patients. These data were obtained at the time of hospital admission for delivery. The comparison analysis revealed apparent differences in demographic and medical factors between the control and pre-eclampsia groups. Individuals in the pre-eclampsia group are generally older with a higher BMI. It is also more common in this group to have a family history of pre-eclampsia, as well as pre-existing hypertension and diabetes, suggesting a strong association with the development of pre-eclampsia. Educational achievements and incomes are noticeably lower in people with pre-eclampsia, suggesting that socioeconomic factors might impact their risks of developing these conditions. Additionally, employment rates are also lower within this population. Patients with pre-eclampsia have a more significant number of prior pregnancies and fewer prenatal appointments, highlighting the significance of proper prenatal care in preventing this condition.

Examining biochemical parameters in both the control and pre-eclampsia groups shows significant differences, such as higher proteinuria and sFlt-1 and reduced PlGF in the pre-eclampsia group, highlighting their importance for diagnosing this condition. Moreover, increased levels of the liver enzymes ALT and AST point to potential liver damage. At the same time, a slight decrease in serum creatinine within the preeclamptic group is clinically significant, signaling renal function alterations. In contrast, similarities in serum uric acid levels and uric acid/creatinine ratios suggest these have less influence than other biomarkers studied. Thus, carefully monitoring these factors remains essential for effectively managing pre-eclampsia and providing timely interventions during complications.

Table 2 shows a higher occurrence of short- and long-term maternal issues in the pre-eclampsia group compared to the control group. The pre-eclampsia group had an elevated rate of severe conditions like eclampsia HELLP syndrome, acute kidney injury, liver dysfunction, placental abruption, and postpartum hemorrhage. These results emphasize the increased risk and seriousness of complications related to pre-eclampsia. It is supported by significant statistical values signifying distinct differences in maternal health outcomes between the two groups. Long-term studies on women’s health indicate that individuals with a history of preeclampsia are at greater risk for chronic hypertension, heart disease, kidney problems, diabetes, and mental health difficulties compared to those without such a history.

Babies born from mothers with pre-eclampsia are more likely to be premature, have a lower birth weight, score lower on the APGAR scale, and require care in the NICU than those born to healthy mothers. Their risks of experiencing respiratory distress syndrome and dying as neonates are also higher. Analysis over a long period (5 years after birth) suggests that babies born to mothers with pre-eclampsia often experience more neuropsychological delays, chronic respiratory issues, behavioral disorders, learning challenges, and frequent infections compared to those in the control group (Table 3).

Table 3 also illustrates that children born to mothers with pre-eclampsia (the PEG) are more likely to be diagnosed with multiple health conditions compared to those born to mothers without pre-eclampsia (the CG). Specifically, 5.10% of the children in the PEG had diagnoses in three or more categories, compared to only 1.46% in the CG. Similarly, 8.16% of the PEG children had diagnoses in two categories versus 2.18% in the CG. These findings suggest that pre-eclampsia significantly increases the risk of children developing multiple, overlapping health issues, highlighting the need for vigilant long-term care and monitoring in these children.

After adjusting for gestational age at birth, the percentages of children with various outcomes in both the PEG and CG have decreased slightly, reflecting that some initial findings were likely influenced by the higher rate of preterm birth in the PEG. However, the data still show that children born to mothers with pre-eclampsia are at a significantly higher risk for these conditions compared to those in the control group, even when accounting for gestational age.

These adjusted findings suggest that while preterm birth does contribute to the observed outcomes, pre-eclampsia itself remains a significant factor in the increased risk of neuropsychological delays, chronic respiratory issues, behavioral disorders, learning difficulties, and frequent infections.

The analysis of the correlation between pre-existing hypertension (HTN) and long-term complications in mothers reveals a significant association. In the PEG, 21.42% of mothers had pre-existing HTN compared to 5.10% in the control group (CG), a statistically significant difference (*p* < 0.0001). In the long term, these mothers had a higher prevalence of chronic HTN (24.48% in the PEG vs. 10.21% in the CG, *p* = 0.0035) and cardiovascular diseases (20% in the PEG vs. 8.02% in the CG, *p* = 0.0072), highlighting an increased risk of developing hypertensive and cardiovascular complications.

Pre-existing diabetes mellitus (DM) was also more frequent in the PEG (16.32%) compared to the control group (8.02%, *p* = 0.0496). This correlates with a higher prevalence of chronic diabetes (15.3% in the PEG vs. 5.10% in the CG, *p* = 0.0082) and kidney diseases (11.22% in the PEG vs. 2.91% in the CG, *p* = 0.0103) in the long term, underscoring the significant impact of pre-existing DM on the long-term health of mothers. These findings suggest that proper management of DM before and during pregnancy is crucial to reducing associated long-term risks.

The impact of pre-existing HTN and DM on children’s health is also significant. Children born to mothers with pre-existing HTN had an increased prevalence of delayed neuropsychological development (15.30% in the PEG vs. 5.1% in the CG, *p* = 0.0082) and chronic respiratory problems (12.24% in the PEG vs. 3.64% in the CG, *p* = 0.0122). Similarly, children of mothers with pre-existing DM had higher rates of learning difficulties (21.42% in the PEG vs. 7.29% in the CG, *p* = 0.0016) and frequent infections (18.36% in the PEG vs. 8.02% in the CG, *p* = 0.0177). These data suggest that mothers’ pre-existing conditions negatively impact the long-term health of their children, highlighting the need for continuous intervention and monitoring to minimize morbidity risks.

## 4. Discussion

This study comprehensively analyzes the significant differences between the control group and those with pre-eclampsia. The data reinforce existing research, which has consistently identified advanced age and elevated BMI as critical risk factors for the development of pre-eclampsia. These findings are particularly significant in public health, highlighting the importance of monitoring and managing these risk factors in pregnant women. Furthermore, the observed presence of a family history of pre-eclampsia adds to the growing body of evidence suggesting a genetic predisposition to the condition. This supports previous research indicating that genetic factors play a role in the transmission and development of pre-eclampsia, making it imperative for future studies to explore the specific genetic markers involved [9,10,11].

The lower levels of education and income within the pre-eclampsia group not only suggest a significant influence of socioeconomic factors but also underscore the complex interplay between social determinants of health and pregnancy outcomes. While prior studies have touched upon this relationship, our research provides additional evidence linking limited access to prenatal care with a heightened risk of complications associated with pre-eclampsia. This is particularly concerning given that prenatal care is essential for the early detection and management of potential health issues during pregnancy. The decrease in employment rates among women in the pre-eclampsia group further highlights the economic struggles these women face, suggesting that pre-eclampsia may have a broader impact on their ability to maintain employment and financial stability during and after pregnancy. These findings emphasize the need for targeted interventions to improve access to healthcare and economic support for vulnerable populations [12,13].

Moreover, our study identifies notable variations in critical biomarkers, such as proteinuria, sFlt-1, and PlGF levels, across different groups. This finding underscores the crucial role these biomarkers play in diagnosing and understanding the pathophysiology of pre-eclampsia. As documented in earlier research, the observed association between decreased levels of sFlt-1 and PlGF with endothelial dysfunction and placental stress is exciting. It suggests that monitoring these biomarkers could be pivotal in identifying women at risk for pre-eclampsia and managing the condition more effectively. Furthermore, the elevated levels of liver enzymes, such as ALT and AST, in the pre-eclampsia group indicate liver damage, which aligns with the development of HELLP syndrome—a severe and life-threatening complication of pre-eclampsia. These findings highlight the importance of early biomarker assessment in preventing the progression of pre-eclampsia to more severe forms [14,15,16,17].

Additionally, our findings on serum creatinine, uric acid levels, and their respective ratios reveal potential alterations in kidney function among women with pre-eclampsia. These results suggest that the kidney damage observed in pre-eclampsia might be more nuanced than previously thought, involving subtle changes that could have long-term implications for renal health. The significant differences in proteinuria levels and urine specific gravity between study groups further corroborate the presence of kidney damage in pre-eclampsia, which is consistent with the existing literature on renal complications in this condition. These findings underscore the need for continuous monitoring of renal function in women with pre-eclampsia to ensure timely and appropriate intervention [18,19,20].

Our study also brings to light the severe maternal complications associated with pre-eclampsia, including eclampsia, HELLP syndrome, acute renal failure, liver dysfunction, and abruption. These conditions, which are more prevalent among women with pre-eclampsia, highlight the critical importance of early and accurate diagnosis, as well as the necessity for prompt medical intervention. The increased risk of such complications in the pre-eclampsia group underscores the urgency of improving clinical practices to manage better and mitigate the adverse outcomes associated with pre-eclampsia. Moreover, our research indicates that infants born to mothers with pre-eclampsia are at a significantly higher risk for premature birth, low birth weight, and NICU admission. These neonatal outcomes are particularly concerning, as they are closely linked to long-term health challenges for the affected infants. The association between pre-eclampsia and adverse neonatal outcomes further emphasizes the need for comprehensive prenatal care that includes strategies for managing high-risk pregnancies [21,22,23,24,25,26,27].

In terms of long-term maternal health, our study reveals a higher prevalence of chronic hypertension, cardiovascular diseases, kidney issues, diabetes, and mental health conditions among women with a history of pre-eclampsia. This suggests a potential link between pre-eclampsia and an increased risk of long-term cardiovascular and metabolic disorders. These findings are significant as they point to the need for ongoing monitoring and management of women who have experienced pre-eclampsia, even after pregnancy. The elevated risks of chronic conditions in these women underscore the potential for pre-eclampsia to have lasting effects on overall health and well-being, necessitating a multidisciplinary approach to care that extends beyond the postpartum period [28,29,30,31].

Finally, our study contributes to the growing body of evidence indicating that children born to mothers with pre-eclampsia are at an increased risk of neuropsychological developmental delays, ongoing respiratory problems, behavioral issues, learning difficulties, and frequent infections. These findings highlight the far-reaching impact of pre-eclampsia, not only on maternal health but also on the long-term health and development of the offspring. The association between prenatal exposure to pre-eclampsia and adverse neurodevelopmental outcomes in children underscores the importance of early intervention and continuous support to address these challenges. Our research suggests that children exposed to pre-eclampsia in utero may benefit from targeted developmental assessments and interventions to mitigate the potential long-term effects on their health and well-being [32,33].

### Strength and Limitation

Our study has several vital strengths. First, it uses a large sample size of 238 patients, which allows for thorough statistical analysis and shows clear contrasts between the control group and those with pre-eclampsia. Second, we examine serum and urinary biomarkers such as sFlt-1, PlGF, ALT, and AST, providing insight into the biochemical changes associated with pre-eclampsia and helping to identify potential signs for diagnosis and monitoring purposes. The research also examines various aspects, including demographic, socioeconomic, and medical factors, as well as immediate and future complications, giving us a comprehensive understanding of the impacts of pre-eclampsia.

The study has some limitations that need to be considered. A significant limitation is the observational nature of this research, which cannot establish a definite causal relationship between pre-eclampsia and the examined complications. Additionally, reliance on self-reported data for variables such as family history and socioeconomic status might introduce reporting bias. Also, data collection was limited to a single medical facility, possibly restricting the findings’ applicability to other groups and locations. Finally, even though it evaluated various biomarkers, not all possible factors affecting the outcome were included, such as genetic and environmental variables.

## 5. Conclusions

Our study identified significant differences between the control and pre-eclampsia groups in demographics, biochemical markers, and maternal and neonatal outcomes. Women with pre-eclampsia were older, had higher BMIs, and more often had a family history of the condition, pre-existing hypertension, and diabetes. They had lower educational and socioeconomic levels and fewer prenatal visits. Biochemical markers like higher proteinuria sFlt-1 and lower PlGF were significant in diagnosing pre-eclampsia. Maternal complications were more frequent, and neonatal outcomes were poorer, including higher risks of preterm birth and developmental issues. These findings emphasize the importance of early monitoring and interventions to effectively manage pre-eclampsia and its consequences, ensuring better health outcomes for mothers and their children.

## Figures and Tables

**Table 1:** Demographic and medical factors.

Factor		PEG (n = 98)	CG (n = 137)	*p*-Value
Demographic and medical factors	Age (years) ^(a)^	30 ± 5	28 ± 4	0.0008 *
	BMI ^(a)^	28.5 ± 3.2	26.5 ± 2.8	<0.0001 **
	Family history of pre-eclampsia ^(b)^	32 (32.65%)	12 (8.75%)	<0.0001**
	Pre-existing hypertension ^(b)^	21 (21.42%)	7 (5.10%)	<0.0001 **
	Diabetes ^(b)^	16 (16.32%)	11 (8.02%)	0.0496 *
	Smokers ^(b)^	24 (24.48%)	22 (16.05%)	0.1090
	Educational level—college graduates ^(b)^	59 (60.20%)	112 (81.75%)	0.0003 **
	Income level—middle income ^(b)^	39 (39.79%)	83 (60.58%)	0.0017 **
	Occupation—employed ^(b)^	49 (50%)	96 (70.07%)	0.0018 **
	Residence—urban ^(b)^	55 (56.12%)	88 (64.23%)	0.2101
	Number of previous pregnancies ^(a)^	3 ± 1	2 ± 1	<0.0001 **
	Prenatal care visits ^(a)^	6 ± 2	8 ± 2	<0.0001 **
Biochemical parameters	Serum creatinine (mg/dL) ^(a)^	0.4754 ± 0.1564	0.5296 ± 0.1123	0.0170 *
	Serum uric acid (mg/dL) ^(a)^	3.966 ± 1.150	3.993 ± 1.140	0.8877
	Serum uric acid to creatinine ratio^(a)^	8.23 ± 1.046	8.46 ± 2.587	0.3787
	PlGF (pg/mL) ^(a)^	51 ± 15.1	152 ± 32	<0.0001 **
	sFlt-1 (pg/mL) ^(a)^	8364 ± 1283	152 ± 32	<0.0001 **
	ALT (U/L) ^(a)^	45 ± 9	22 ± 4	<0.0001 **
	AST (U/L) ^(a)^	51 ± 11	24 ± 3	<0.0001 **
Urine test	Proteinuria (mg/dL) ^(a)^	331 ± 38	51 ± 9	<0.0001 *
	Urine output (mL/day) ^(a)^	832 ± 137	1532 ± 210	<0.0001 *
	Urine specific gravity ^(a)^	1.025 ± 0.003	1.015 ± 0.002	<0.0001 *

^(a)^ mean ± std. dev.; ^(b)^ Observed frequency (percentage); Chi-squared statistical test; * *p*-value< 0.05; ** *p*-value < 0.01.

**Table 2:** Short- and long-term maternal complications in CG and PEG.

Complication		PEG	CG	*p*-Value
Short-term complications	Eclampsia ^(a)^	5 (5.1%)	0 (0%)	0.0077 **
	HELLP Syndrome ^(a)^	7 (7.14%)	0 (0%)	0.0015 **
	Acute Kidney Injury ^(a)^	9 (9.18%)	1 (0.72%)	0.0016 **
	Liver Dysfunction ^(a)^	11 (11.22%)	3 (2.18%)	0.0039 **
	Placental Abruption ^(a)^	6 (6.12%)	0 (0%)	0.0034 **
	Postpartum Hemorrhage ^(a)^	13 (13.26%)	4 (2.91%)	0.0026 **
Long-term complications	Chronic Hypertension ^(a)^	24 (24.48%)	14 (10.21%)	0.0035 **
	Cardiovascular Disease ^(a)^	19 (20%)	11 (8.02%)	0.0072 **
	Kidney Disease ^(a)^	11 (11.22%)	4 (2.91%)	0.0103 *
	Diabetes ^(a)^	15 (15.3%)	7 (5.10%)	0.0082 **
	Mental Health Disorders ^(a)^	9 (9.18%)	15 (10.94%)	0.6610

^(a)^ Observed frequency (percentage); Chi-squared statistical test; * *p*-value< 0.05; ** *p*-value < 0.01.

**Table 3:** Neonatal outcomes in CG and PEG.

Outcome			PEG	CG	*p*-Value
Neonatal outcomes	Preterm Birth ^(a)^		29 (29.59%)	14 (10.21%)	<0.0001 **
	Low Birth Weight ^(a)^		24 (24.48%)	7 (5.10%)	<0.0001 **
	NICU Admission ^(a)^		21 (15.32%)	7 (5.10%)	<0.0001 **
	Respiratory Distress Syndrome ^(a)^		11 (11.22%)	3 (2.18%)	<0.0001 **
	Neonatal Mortality ^(a)^		4 (2.9%)	0 (0%)	<0.0001 **
	Apgar Scores ^(b)^		8.460 ± 0.6675	8.945 ± 0.5581	<0.0001 **
	Gestational weeks ^(b)^		35.43 ± 2.039	37.38 ±1.022	<0.0001 **
	Baby birth weight ^(b)^		2995 ± 580.8	3373 ± 457.5	<0.0001 **
Long-term outcomes	Delayed Neuropsychological Development ^(a)^	Before Correcting for GA ^(c)^	15 (15.30%)	7 (5.1%)	0.0082 **
		After Correcting for GA ^(c)^	10 (10.20%)	6 (4.38%)	0.0221
	Chronic Respiratory Problems ^(a)^	Before Correcting for GA ^(c)^	12 (12.24%)	5 (3.64%)	0.0122 *
		After Correcting for GA ^(c)^	8 (8.16%)	4 (2.92%)	0.0315
	Behavioral Disorders ^(a)^	Before Correcting for GA ^(c)^	10 (10.20%)	4 (2.91%)	0.0201 *
		After Correcting for GA ^(c)^	7 (7.14%)	3 (2.19%)	0.0417
	Learning Difficulties ^(a)^	Before Correcting for GA ^(c)^	21 (21.42%)	10 (7.29%)	0.0016 **
		After Correcting for GA ^(c)^	15 (15.31%)	8 (5.84%)	0.0113
	Frequent Infections ^(a)^	Before Correcting for GA ^(c)^	18 (18.36%)	11 (8.02%)	0.0177 *
		After Correcting for GA ^(c)^	13 (13.27%)	9 (6.57%)	0.0248
Diagnoses in 3 or more categories			5 (5.10%)	2 (1.46%)	0.1063
Diagnoses in 2 categories			8 (8.16%)	3 (2.18%)	0.0326

^(a)^ Observed frequency (percentage); Chi-squared statistical test; ^(b)^ mean ± std. dev.; * *p*-value< 0.05; ** *p*-value < 0.01; ^(c)^ Gestational age.

## Data Availability

The raw data supporting the conclusions of this article will be made available by the authors upon request.
